# Photoresponsive Zinc‐Based Batteries

**DOI:** 10.1002/smsc.202300034

**Published:** 2023-07-21

**Authors:** Xiaofeng Lin, Zilong Liu, Xiaotong Wang, Ping Li, Dingshan Yu

**Affiliations:** ^1^ School of Chemical Engineering and Light Industry Guangdong University of Technology Guangzhou 510006 P. R. China; ^2^ School of Chemistry and Chemical Engineering Chongqing University of Science & Technology Chongqing 401331 China; ^3^ Key Laboratory for Polymeric Composite and Functional Materials of Ministry of Education Sun Yat-Sen University Guangzhou 510275 P. R. China

**Keywords:** energy conversion and storage, photoelectrodes, photoresponsive batteries, zinc-based batteries

## Abstract

Photoresponsive batteries are an innovative technology that combines conversion and storage of solar energy, providing a potential solution for large‐scale utilization of solar energy while reducing the reliance on traditional energy storage devices to meet the growing demand for energy. In search of more resource‐saving alternatives to lithium‐ion batteries, researchers are turning to zinc, which is abundant and environmentally friendly due to its recyclability. Zinc batteries offer high energy density and aqueous stability, making them a popular choice among researchers. This review summarizes the recent progress in photoresponsive zinc‐based batteries. First, the photoresponsive zinc‐based batteries are categorized into two groups: photoresponsive zinc‐ion batteries and photoresponsive zinc–air batteries. The discussion then focuses on various photoelectrode designs, including some interesting strategies. Finally, the technical challenges associated with photoresponsive zinc‐based batteries are summarized and discussed, and future research directions are proposed.

## Graphical abstract

Photoresponsive zinc‐based batteries with excellent energy storage properties, which are the integration of zinc‐based batteries with solar energy, are significant advancements toward the future utilization of solar energy.

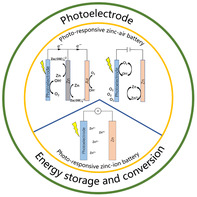

## 1 Introduction

As human society and technology continue to advance, the demand for energy is increasing at an unprecedented rate. However, the use of fossil fuels has resulted in significant environmental issues, such as air pollution and global warming, which have raised concerns regarding their long‐term sustainability. Therefore, there has been a growing interest in the development of clean energy sources.^[^ 1, 2 ^]^ Among these clean energy sources, solar energy has been identified as the most promising sustainable energy source, with a potential total energy capacity of up to 1 × 10^5^ TW h^−1^, which is sufficient to meet the global annual energy demand of 16 TW.^[^ ^3^ ^]^ Nevertheless, owing to the intermittent nature of solar energy caused by the daily alternation of day and night, more effective storage of solar energy should be carried out for utilization during nonilluminated periods.^[^ 4, 5, 6 ^]^

Solar energy can be converted in various forms, including photovoltaic conversion, photothermal conversion and photochemical conversion, etc.^[^ ^7^ ^]^ After solar energy conversion, one of the most effective storage methods is through electrochemical storage, which is an essential technology for future carbon‐neutral power systems.^[^ 8, 9 ^]^ Among the various electrochemical storage technologies, rechargeable batteries offer a solution to the increasing demand for energy storage in both mobile and stationary applications, benefitting from their ability to effectively convert between chemical energy and electrical energy. Therefore, the integration of solar energy with advanced secondary batteries for efficient energy storage and conversion is crucial.^[^ 10, 11, 12 ^]^ Solar energy can not only be stored as electrochemical energy but can also be used as a responsive module in a battery. By combining rechargeable batteries with solar energy response, both charge and discharge performance of such photoresponsive batteries can be effectively improved; meanwhile, the utilization efficiency of solar energy can also be promoted. The principle of photoresponsive batteries is that sunlight irradiates the photoelectrode to generate electron–hole pairs. Next, the electrons are introduced into the external circuit to produce photocurrent, while oxidizing holes are left in the photoelectrode for redox reactions. The generation of photocurrent can reduce the use of external voltage (photoassisted battery) or even eliminate the need for external voltage (light self‐charging battery), which not only increases the utilization of solar energy but also saves energy consumption.

Zinc, a common element found in the Earth's crust, is characterized by low manufacturing cost and ease of recovery through electrochemical or thermochemical processes to obtain zinc or zinc oxide. Despite the relatively mature technology of lithium‐ion batteries, the disadvantages of lithium‐ion batteries, such as the electrolyte flammability, high cost, and potential toxicity, are still difficult to overcome.^[^ 13, 14 ^]^ Due to the high stability of zinc‐based batteries (e.g., zinc‐ion batteries and zinc–air batteries), they could be promising energy storage solutions, making zinc an ideal choice as battery anodes.^[^ 15, 16, 17, 18, 19 ^]^ Both the zinc‐ion batteries and zinc–air batteries have high theoretical capacity. Moreover, zinc–air batteries (1086 Wh kg^−1^) can even store several times more capacity than traditional lithium‐ion batteries.^[^ 15, 16, 17, 18, 19 ^]^ Thus, the integration of zinc‐based batteries with solar energy, which can obtain photoresponsive zinc‐based batteries with excellent energy storage properties, is a significant advance toward the future utilization of solar energy.^[^ ^20^ ^]^

Among the research of photoresponsive zinc‐based batteries, the design of the photoelectrode has garnered much attention as a popular research topic.^[^ ^21^ ^]^ Notably, the material and structure of photoelectrode have the most significant impact on the performance of the zinc‐based photoresponsive battery. The main challenge in photoelectrode design is to improve light absorption while avoid recombination of photongenerated carriers. To address this challenge, researchers have employed various approaches. They have developed a series of photoresponsive materials as a single‐layer semiconductor in the photoelectrode to enhance solar energy utilization, such as VO_2_, MnO_2_, MoS_2_, etc. Also, adjusting the bandgap width of photoresponsive materials can be an advisable strategy to increase the light absorption.^[^ ^22^ ^]^ In addition, the modification of photoresponsive materials is one of the vital aspects of the research. The absorption of sunlight can be improved more effectively in the photoelectrode, which is attributed to the enhancement of carrier separation's effect by doping different inorganic elements (Co/Ni, etc.) into the photoresponsive materials.^[^ 23, 24 ^]^ Moreover, the different heterojunction design (e.g., Z‐type/S‐type, etc.) in the photoelectrode can improve the efficiency of exciton separation and increase light absorption.^[^ 25, 26 ^]^ Besides, some researches have focused on improving the conductivity and stability of the photoresponsive materials, optimizing the photoelectrode's structures and morphology, and enhancing the reaction kinetics to improve the light response in photoelectrode design.^[^ 27, 28, 29, 30, 31 ^]^ Therefore, the design of photoresponsive zinc‐based batteries is a complex and multifaceted problem that requires further improvement in battery performance.

In this review, the comprehensive overview of recent developments for photoresponsive zinc‐based batteries has been summed up, which mainly focuses on the selection and construction strategies of photoelectrodes. First, the working principle and mechanism of photoresponsive zinc‐based batteries have been demonstrated. It then proceeds to summarize recent design strategies employed in the development of photoresponsive zinc‐based batteries, including photoresponsive zinc‐ion batteries and photoresponsive zinc–air batteries. Additionally, several interesting design strategies of other optimized strategies for photoresponsive zinc‐based batteries are introduced in a succinct manner. Finally, the discussion of the opportunities and challenges facing photoresponse zinc‐based batteries in practical applications is concluded, with the objective of inspiring more researchers to explore novel approaches to design highly efficient photoresponsive batteries.

## 2 Configurations and Principles

This section aims to provide a succinct introduction to the working principle of photoresponsive zinc batteries, as well as the photoresponsive mechanism and a brief overview of several commonly used heterojunctions.

### 2.1 Basics of Photoresponsive Mechanism

In general, the utilization of the solar energy in photoresponsive batteries could be considered as a photoelectric and photothermal effect, while the photoelectric effect is investigated more than photothermal effect. In the following part, the mechanisms of photoelectric and photothermal effects in photoresponsive batteries are briefly described.

**Figure 1:**
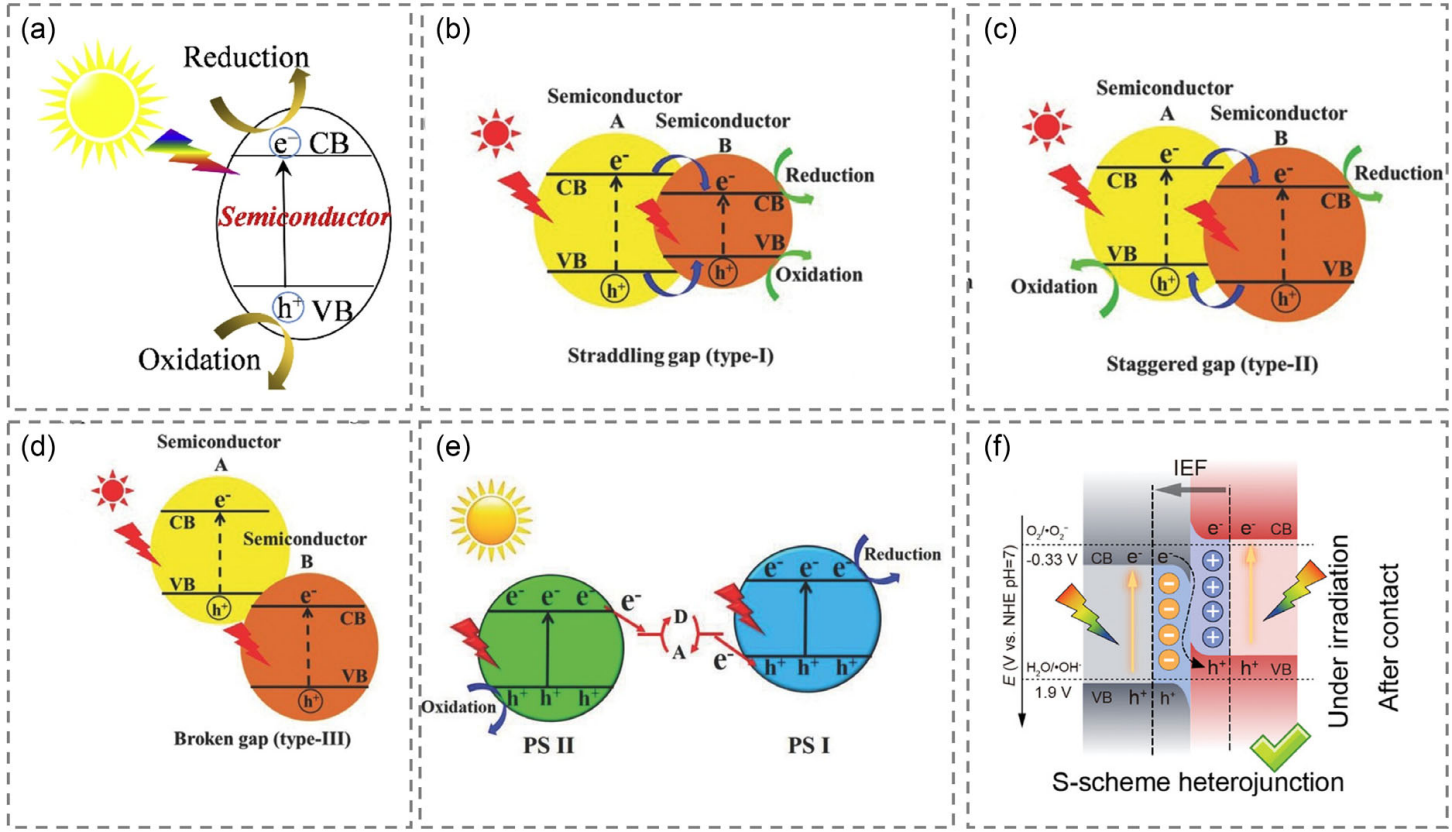
a) The working mechanism of photoresponsive semiconductors. Reproduced with permission.^[^ ^36^ ^]^ Copyright 2017, Elsevier. b) Type‐I heterojunction. Reproduced with permission.^[^ ^35^ ^]^ Copyright 2017, Wiley‐VCH. c) Type‐II heterojunction. Reproduced with permission.^[^ ^35^ ^]^ Copyright 2017, Wiley‐VCH. d) Type‐III heterojunction. Reproduced with permission.^[^ ^35^ ^]^ Copyright 2017, Wiley‐VCH. e) Z‐scheme heterojunction. Reproduced with permission.^[^ ^35^ ^]^ Copyright 2017, Wiley‐VCH. f) S‐scheme heterojunction. Reproduced with permission.^[^ ^26^ ^]^ Copyright 2022, Wiley‐VCH.

Photoresponsive battery is a new type of energy conversion device containing the photoelectrode. Unlike conventional batteries, photoresponsive batteries can rely on the ability of photoelectrodes to collect/convert photons, achieving partial or complete replacement of external charging energy and enhanced output energy. When a photoresponsive material in the photoelectrode absorbs photons with energy equal to or greater than its bandgap (*E*g), electrons in the filled valence band (VB) become excited to the empty conduct band (CB), creating holes in the VB. The optical absorption and redox properties of the photoresponsive material are determined by the value of *E*_g_ and the position of the CB and VB band edges(**Figure** **1**a).^[^ ^32^ ^]^ Although many semiconductor materials can be used as photoresponsive materials.^[^ 33, 34 ^]^ photoelectrode reactions based on various photoresponsive materials can still be comparatively straightforward to comprehend. Five steps are involved in the photoelectrode reactions based on photoresponsive materials: 1) light absorption by the semiconductor, 2) formation of photogenerated electron–hole pairs, 3) migration and recombination of electron–hole pairs, 4) adsorption and desorption of reactants and products, and 5) oxidation–reduction reactions on the semiconductor surface. Generally, the oxidation ability is stronger when the valence band top is more positive, while the reduction ability is stronger when the conduction band bottom is more negative.^[^ ^35^ ^]^ The ability of photoelectrons or holes to migrate is more favorable for oxidation–reduction reactions when the valence or conduction band has better off‐diagonal character.^[^ ^36^ ^]^ Meanwhile, this is the original idea of photoelectrode design based on a single‐layer semiconductor. The single‐layer semiconductor mainly could be inorganic photoresponsive material, such as TiO_2_, ZnS, Co_3_O_4_ NiCo_2_S_4_, BiVO_4_ or α‐Fe_2_O_3_, etc., which have been reported to be effective, stable, and integrated metal–air photoelectrode materials. In addition, the optimization idea of single‐layer semiconductors in the photoelectrode is to improve the photochemical activity by modifying the surface of semiconductor materials, so as to make the photoelectric conversion efficiency higher.

However, the photoelectrode based on a single‐layer semiconductor is difficult to achieve high photoconversion efficiency due to the relatively low separation efficiency of photogenerated carriers. The internal electric field (IEF) introduced by the construction of heterojunction can effectively promote the separation and diffusion of photogenerated carriers. Therefore, the low efficiency of charge separation can be effectively solved by constructing heterojunction semiconductor systems.^[^ 37, 38 ^]^ Several common heterojunction types, such as interface gap type (I type) (Figure 1b), alternating gap type (II type) (Figure 1c), and fracture gap type (III type) (Figure 1d), as well as some new Z‐type (Figure 1e) and S‐type (Figure 1f) heterojunctions, are summarized here. In I‐type heterojunction, the CB of semiconductor A is higher than that of semiconductor B and the VB of semiconductor A is lower than that of semiconductor B (Figure 1b). Under illumination, electrons and holes accumulate respectively at the CB and VB energy levels of semiconductor B. Since electrons and holes are both concentrated on the same semiconductor, the electron–hole pair cannot be effectively separated. In addition, the oxidation–reduction reaction occurs on the semiconductor with a lower oxidation–reduction potential, significantly reducing the oxidation–reduction ability of the heterojunction. In II‐type heterojunction, photogenerated electrons transfer to semiconductor B under illumination, while photogenerated holes migrate to semiconductor A, resulting in the separation of electron–hole pairs in space. However, like I‐type heterojunction, the oxidation–reduction ability of II‐type heterojunction will be reduced. The III‐type heterojunction structure is similar to that of the II‐type heterojunction, but the alternating gap between the III‐type heterojunction becomes so extreme that the bandgap does not overlap. Therefore, III‐type heterojunction cannot cause electron–hole migration and separation between the two semiconductors and is not suitable for enhancing the separation of electron–hole pairs. Among the conventional heterojunctions mentioned above, II‐type heterojunction is the most effective conventional heterojunction in terms of improving photogenic charge separation ability.^[^ ^35^ ^]^ The construction of a Z‐type heterojunction can lead to an effective interfacial electron transfer channel with a large IEF, which can result in efficient and stable photoresponsive performance.^[^ ^39^ ^]^ The Z‐type heterojunction is characterized by the spatial separation of photogenerated electrons and holes across two semiconductors with different band structures and is achieved by precisely regulating the interfacial interaction. The electrons from the conduction band of one semiconductor are transferred to another with a higher reduction potential, while the holes from the valence band are transferred to the other semiconductor with a higher oxidation potential. This separation allows for the best oxidation–reduction ability. However, traditional Z‐type structures are limited to the liquid phase, which restricts their use in photoelectrode. In contrast, S‐type heterojunctions form an IEF between two semiconductors, allowing low oxidation–reduction position heterojunction to be directly composited. This preserves electron–hole pairs in a higher oxidation–reduction state and enhances the photogenic charge separation ability.^[^ 26, 40, 41, 42 ^]^

**Figure 2:**
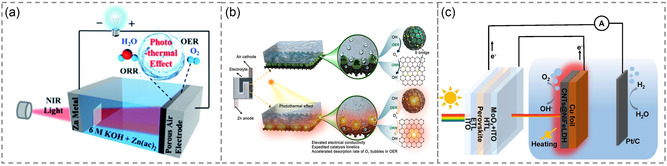
a) Synthetic strategy of the photothermal‐assisted ZAB. Reproduced with permission.^[^ ^44^ ^]^ Copyright 2021, Royal Society of Chemistry. b) Assembled photoresponsive zinc–air batteries. Reproduced with permission.^[^ ^45^ ^]^ Copyright 2023, American Chemical Society. c) Integrated semitransparent perovskite solar battery and electrocatalytic system. Reproduced with permission.^[^ ^46^ ^]^ Copyright 2023, Elsevier.

The efficiency of the photoresponsive battery can be enhanced by incorporating thermal energy, which is known as photothermal effect. This approach utilizes the synergistic effect of light and heat to promote chemical reactions, with the absorbed energy increasing the energy of the reactants and thus facilitating the reaction. The process of thermally assisted photocatalysis involves electron excitation and hot carrier generation. The heat energy generated by the photoresponsive materials can reduce the activation energy of the reaction, thus improving the light conversion efficiency.^[^ ^43^ ^]^ In a study by Zhang et al.,^[^ ^44^ ^]^ a bifunctional electrocatalyst (Co_3_O_4_/N‐rGO) was employed for N‐doped reduction of Co_3_O_4_ nanoparticles in GO as the active material (**Figure** **2**a). The photothermal effect of Co_3_O_4_/N‐rGO under illumination induces local heating, creating more active sites and enhancing electrical conductivity, thereby resulting in improved overall catalytic performance. In addition to doping deposition, spherical catalysts are known to exhibit good photothermal response. The spherical cavity is capable of efficiently storing the heat generated during the photothermal conversion process while reducing heat exchange with the environment, resulting in better utilization of heat energy (Figure 2b).^[^ ^45^ ^]^ Furthermore, an integrated approach of connecting solar batteries and thermal response catalysts via external circuits has been proposed to develop dual‐function photothermal catalysts (Figure 2c).^[^ ^46^ ^]^ This type of catalyst requires only a single light/thermal response, and its design is more flexible and stable.

Compared with the traditional battery systems, the photoresponsive battery system not only reduces energy loss, but also improves the efficiency of the battery using solar energy for photoelectric/photothermal conversion. However, the photoelectric effect is reported more than the photothermal effect in photoresponsive zinc‐based batteries, which is more of an auxiliary means. Therefore, the focus of this review is on the design of photoelectrode, while the part of photothermal effect will only be briefly introduced.

### 2.2 Basics of Photoresponsive Zinc‐Ion Batteries

**Figure 3:**
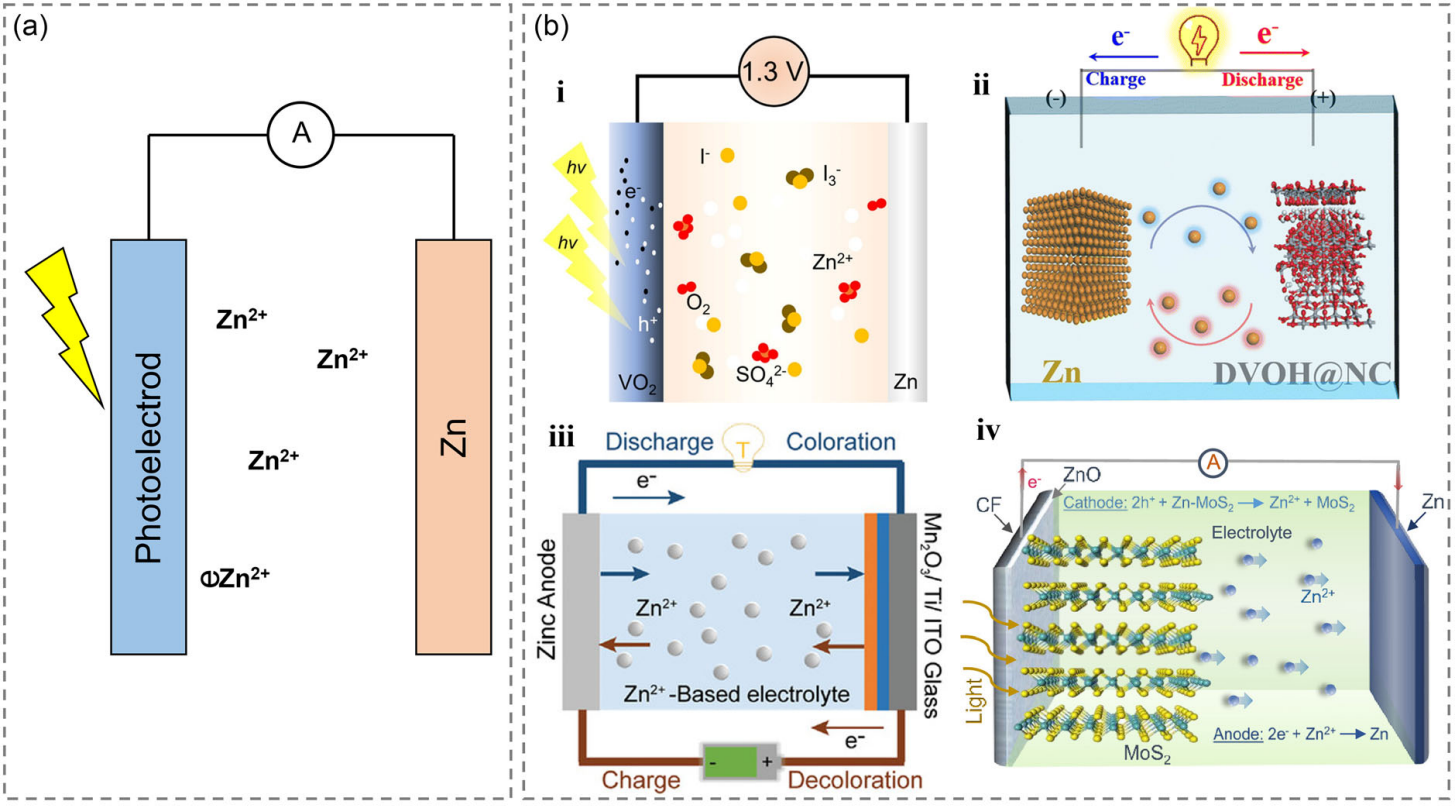
a) Photoresponsive zinc‐ion battery general type. b) Schematic diagram of photoresponsive Zn‐ion batteries with: i) VO_2_ photoelectrode. Reproduced with permission.^[^ ^52^ ^]^ Copyright 2022, Elsevier. ii) DVOH@NC photoelectrode. Reproduced with permission.^[^ ^62^ ^]^ Copyright 2022, Royal Society of Chemistry. iii) Mn_2_O_3_/Ti/ITO photoelectrode, Reproduced with permission.^[^ ^76^ ^]^ Copyright 2021, Wiley‐VCH, and iv) MoS_2_ photoelectrode. Reproduced with permission.^[^ ^77^ ^]^ Copyright 2021, American Chemical Society.

Zinc‐metal electrodes possess several favorable attributes such as multifunctionality, high‐energy density, and cost‐effectiveness, which make them attractive for battery chemistry.^[^ 47, 48, 49, 50 ^]^ However, zinc‐based batteries can be classified as alkalinity, neutrality, or acidity depending on the electrolyte, with different charge–discharge reactions, which is reflected in the different designs of zinc electrodes and electrolytes. But for most zinc‐based batteries, especially photoresponsive zinc‐based batteries, alkaline electrolytes are the most important (**Figure** **3**a).^[^ ^51^ ^]^ Therefore, the use of distinct photoresponsive materials, such as VO_2_ (Figure 3b‐i), DVOH@NC (Figure 3b‐ii), Mn_2_O_3_/Ti/ITO (Figure 3b‐iii), MoS_2_ (Figure 3b‐iv), etc., or unique structural designs of the photoelectrode may be required. During the charging process, photons are absorbed by the photoelectrode, producing electron–hole pairs. The holes are utilized to extract zinc ions, and the electrons flow through the external circuit, reducing zinc ions from the anode to zinc. In a photoresponsive zinc‐ion battery, the photoelectrode typically serves as the cathode. The discharge process is the inverse of the charging process, whereby the anode produces zinc ions, and the photoelectrode stores zinc ions. In alkaline batteries, the charge–discharge reaction can be expressed as follows (“M” is defined as various photoresponsive materials).

Charge

 Photoelectrode

(1) $$\;\text{Photoelectrode}\to e^{-}{\;+\text{ h}}^{+}$$

(2) $$\;{\text{Zn}-\text{M }+\;2h}^{+}\to {\text{Zn}}^{2+}\;+\text{ M}$$

 Anode

(3) $$\;{\text{ZnO }+\;2\text{OH}}^{-}{\;+\text{ H}}_{2}O\to {\text{Zn}(\text{OH})}_{4}^{2-}$$

(4) $$\;{\text{Zn}(\text{OH})}_{4}^{2-}{\;+\;2e}^{-}\to {\text{Zn }+\;4\text{OH}}^{-}$$

Discharge

 Photoelectrode

(5) $$\;{\text{Zn}}^{2+}\;+\text{ M}\to \text{Zn}-M$$

 Anode

(6) $$\;{\text{Zn }+\;4\text{OH}}^{-}\to {\text{Zn}(\text{OH})}_{4}^{2-}{\;+\;2e}^{-}$$

(7) $$\;{\text{Zn}(\text{OH})}_{4}^{2-}\to {\text{ZnO }+\text{ OH}}^{-}{\;+\text{ H}}_{2}O$$

### 2.3 Basics of Photoresponsive Zinc–Air Batteries

**Figure 4:**
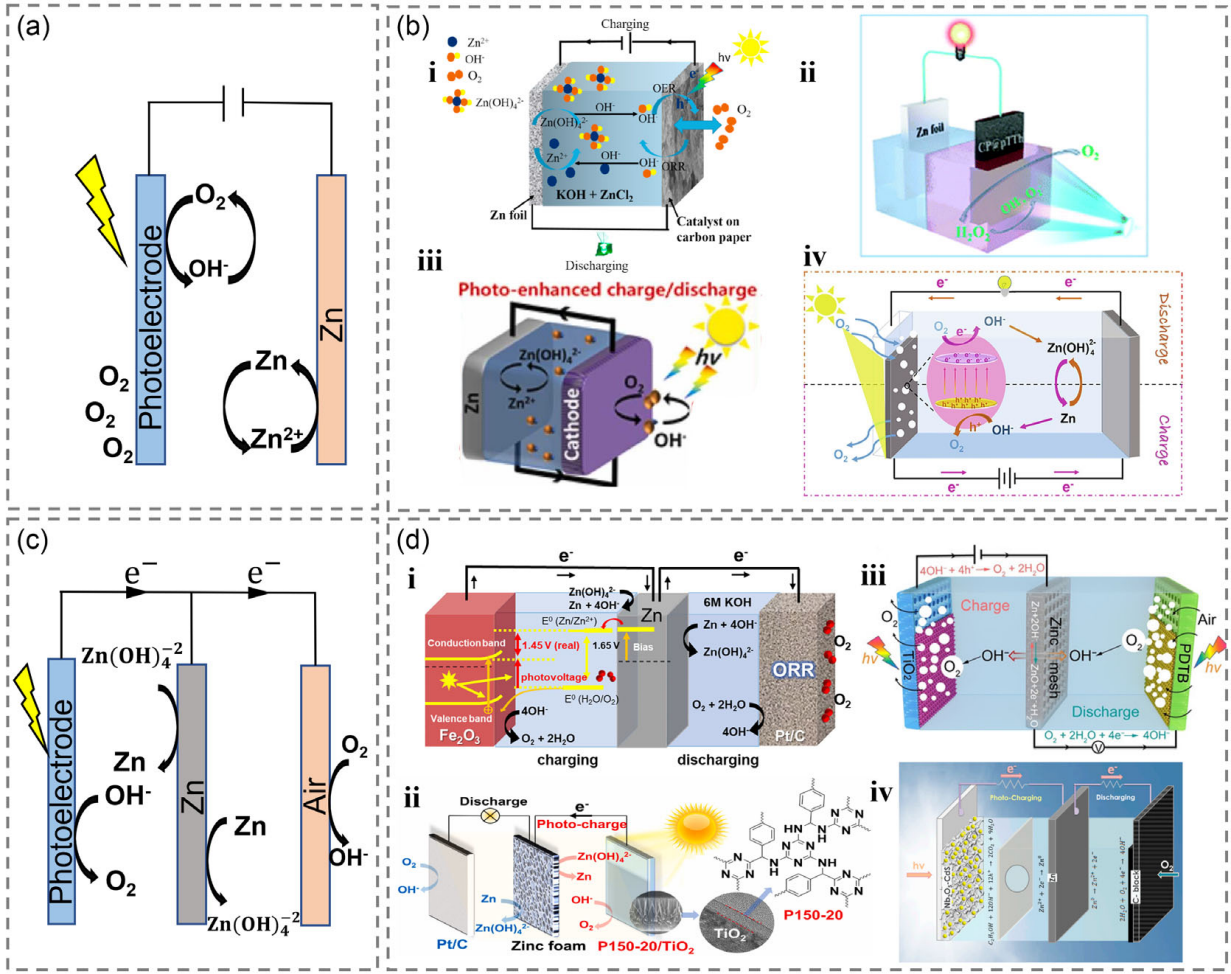
a) Two‐electrode photoresponsive zinc empty battery in general. b) Schematic diagram of two‐electrode photoresponsive Zn–air batteries with difunctional photoelectrodes of: i) d‐MnO_2_. Reproduced with permission.^[^ ^66^ ^]^ Copyright 2019, Royal Society of Chemistry. ii) CP@pTTH. Reproduced with permission.^[^ ^67^ ^]^ Copyright 2022, Elsevier. iii) g‐C_3_N_4_/CuZIF‐67. Reproduced with permission.^[^ ^70^ ^]^ Copyright 2022, Elsevier, and iv) ZnO/Cu_2_O(CuO). Reproduced with permission.^[^ ^72^ ^]^ Copyright 2021, Elsevier. c) Three‐electrode photoresponsive zinc empty battery in general. d) Schematic diagram of three‐electrode photoresponsive Zn–air batteries with photoelectrode of: i) Fe_2_O_3_. Reproduced with permission.^[^ ^64^ ^]^ Copyright 2022, Elsevier. ii) PDTB. Reproduced with permission.^[^ ^65^ ^]^ Copyright 2020, Wiley‐VCH. iii) P150‐20/TiO_2_. Reproduced with permission.^[^ ^78^ ^]^ Copyright 2021, American Chemical Society, and iv) Nb_2_O_5_/CdS. Reproduced with permission.^[^ ^79^ ^]^ Copyright 2022, MDPI.

Photoresponsive zinc–air batteries incorporate a semiconductor photoelectrode into an air electrode, leading to a substantial reduction in the charge potential when exposed to sunlight.^[^ ^52^ ^]^ In conventional metal–air batteries, the cathode primarily mediates the electrochemical reaction pathway based on its inherent chemical structure and reduces the energy barrier.^[^ 53, 54, 55, 56 ^]^ Due to the limited chemical driving force of cathode reaction, the discharge/charge voltage of such batteries is typically much lower/higher than the theoretical equilibrium voltage, resulting in a large overpotential. In contrast, in the photocoupled reaction pathway, charging and discharging voltages are much lower than in the dark and can even break the limit of equilibrium potential. This is attributed to the involvement of photogenerated electron–holes in the cathodic reaction. Consequently, photoresponsive batteries can achieve higher round‐trip efficiencies than traditional devices.^[^ ^57^ ^]^ In the alkaline state, the chemical equation for the charge–discharge reaction of a bifunctional photoresponsive zinc–air battery (**Figure** **4**a,b) is presented below.

Charge

 Photoelectrode

(8) $$\;\text{Photoelectrode}\to e^{-}{+h}^{+}$$

(9) $$\;{4\text{OH}}^{-}{+4h}^{+}\to O_{2}{+2H}_{2}O$$

 Anode

(10) $$\;{\text{ZnO }+\;2\text{OH}}^{-}{\;+\text{ H}}_{2}O\to {\text{Zn}(\text{OH})}_{4}^{2-}$$

(11) $$\;{\text{Zn}(\text{OH})}_{4}^{2-}{\;+\;2e}^{-}\to {\text{Zn }+\;4\text{OH}}^{-}$$

Discharge

 Photoelectrode

(12) $$\;O_{2}{\;+\;2H}_{2}{\text{O }+\;4e}^{-}\to {4\text{OH}}^{-}$$

 Anode

(13) $$\;{\text{Zn}\;+\;4\text{OH}}^{-}\to \text{Zn}{\left(\text{OH}\right)}_{4}^{2-}{\;+\;2e}^{-}$$

(14) $$\;{\text{Zn}(\text{OH})}_{4}^{2-}\to {\text{ZnO }+\;2\text{OH}}^{-}{\;+\text{ H}}_{2}O$$

The reaction principle of the three electrodes (Figure 4c,d) of the photoresponsive zinc–air battery is similar to the reaction described above. Specifically, the photoelectrode generates holes and converts OH^−^ into oxygen during charging, whereas the air electrode converts oxygen into OH^−^ during discharge. Notably, the charge and discharge processes of the three‐electrode zinc–air battery are analogous to those of the two‐electrode battery. Traditional rechargeable zinc–air batteries suffer from sluggish oxygen evolution reaction (OER) kinetics, which is a significant drawback. Photoresponsive charging, on the other hand, can reduce the charging voltage, quicken OER through photoinduced oxidation reaction, and enhance the reaction kinetics and energy efficiency.^[^ ^17^ ^]^

## 3 Photoresponsive Zinc‐Based Batteries

Photoresponsive zinc‐based batteries, which respond to light stimuli, have gained significant attention in the field of energy storage due to their inherent safety, cost‐effectiveness, and ecofriendliness. Recent advancements in high‐performance electrode materials, electrolyte systems, and mechanism research have significantly improved the electrochemical performance of these batteries. This section presents an overview of recent design strategies for photoresponsive zinc‐based batteries, encompassing various aspects of photoelectrode design, such as selection, modification, and structural construction of photoresponsive materials, electronic transport layer, and other innovative designs (**Table** **1** and **2**).

**Table 1:** Configurations and properties of representative photoresponsive zinc‐ion batteries

Photoelectrode	Electrolytes	Capacity	Cycle	Capacity retention	Capacity in light [mAh g^−1^]	Capacity in dark [mAh g^−1^]	Refs.
VO_2_	ZnSO_4_	206 mAh g^−1^ (0.3 A g^−1^)	2000 (2 A g^−1^)	96%	200 (0.3 A g^−1^)	150 (0.3 A g^−1^)	[62]
V_2_O_5_	Zn(CF_3_SO_3_)_2_	370 mAh g^−1^ (50 mA g^−1^)	500 (50 mA g^−1^)	≈60%	370 (50 mA g^−1^)	190 (50 mA g^−1^)	[58]
VO_2_	Zn(CF_3_SO_3_)_2_	315 mAh g^−1^ (200 mA g^−1^)	1000 (1 A g^−1^)	90%	312 (0.2 A g^−1^)	285 (0.2 A g^−1^)	[60]
VO_2_	Zn(CF_3_SO_3_)_2_	432 mAh g^−1^ (200 mA g^−1^)	500 (1 A g^−1^)	73%	432 (0.2 A g^−1^)	367 (0.2 A g^−1^)	[61]
MoS_2_	Zn(CF_3_SO_3_)_2_	340 mAh g^−1^ (100 mA g^−1^)	200 (500 mA g^−1^)	82%	340 (0.1 A g^−1^)	245 (0.1 A g^−1^)	[59]

**Table 2:** Configurations and properties of representative photoresponsive zinc–air batteries

Photoelectrode		Electrolytes	Capacity	Cycle	Charge voltage	Performance in light	Performance in dark	Refs.
Photoelectrode	Current collectors							
C_4_N/CP	–	KOH	519.1 mAh g^−1^ (2.5 mA cm^−2^)	50	1.35 V	1.72 V (*V*_c_)a)	1.28 V (*V*_c_)a)	[63]
PDTB/TiO_2_	CP	KOH	–	33	0.59 V	–	–	[65]
g‐C_3_N_4_/CuZIF‐67	CP	KOH/Zn(Ac)_2_	781.7 mAh g^−1^ (10 mA cm^−2^)	1000	1.99 V	781.9 mAh g^−1^ (10 mA g^−1^)	731.9 mAh g^−1^ (10 mA g^−1^)	[70]
Co‐doped d‐MnO_2_	CP	KOH/ZnCl_2_	685.45 mAh g^−1^ (5 mA cm^−2^)	–	–	1.12 V (Δ*E*)d)	1.05 V (Δ*E*)d)	[67]
PTTH	CP	KOH	–	64 h	2.00 V	1.28 V (*V*_d_)b)	1.78 V (*V*_d_)b)	[68]
ZnO/CuO (Cu_2_O)	CP	KOH/Zn(Ac)_2_	333.5 mAh g^−1^ (0.2 mA cm^−2^)	22 h	1.50 V	0.72 V (*V*_oc_)c)	0.9 V (*V*_oc_)c)	[72]
C4N@TiO_2_NR	CP	KOH	–	120	–	–	–	[26]
(*α* + *δ*)‐MnO_2_	CP	KOH/ZnCl2	710 mAh g^−1^	19 h	–	2.23 V (Δ*V*)e)	2.06 V (Δ*V*)e)	[73]
*α*‐Fe_2_O_3_	CP	KOH/Zn(Ac)_2_	598.7 mAh g^−1^ (0.5 mA cm^−2^)	50 h	≈1.42 V	1.96 V (*V*_c_)a)	1.20 V (*V*_c_)a)	[52]
BiVO_4_	CP	KOH/Zn(Ac)_2_	238.5 mAh g^−1^ (0.5 mA cm^−2^)	–	≈1.20 V	1.96 V (*V*_c_)a)	1.42 V (*V*_c_)a)	[52]
Ni_12_P_5_@NCNT	–	KOH/Zn(Ac)_2_	610 mAh g^−1^	320	1.90 V	0.82 V (Δ*E*)d)	0.80 V (Δ*E*)d)	[80]
NiCo_2_S_4_	FTO	KOH/Zn(Ac)_2_	610 mAh g^−1^ (2 mA cm^−2^)	–	1.91 V	0.82 V (voltage gap)	0.60 V (voltage gap)	[69]
MSAE	CP	PVA/PEO/KOH gel	1.1 mAh cm^−2^ (0.2 mA cm^−2^)	150	–	1.96 V (*V*_c_)a)	1.88 V (*V*_c_)a)	[75]

a) *V*_c_: Charge voltage;

b) *V*_d_: Discharge voltage;

c) *V*_oc_: Open‐circuit voltage;

d) Δ*E*: The oxygen electrode activity parameter;

e) Δ*V*: The difference in voltage between charging and discharging.

### 3.1 Photoresponsive Zinc‐Ion Batteries

The photoelectrode serves the primary function of photon absorption and electron–hole pair generation. The bandgap width of different materials is different, leading to the absorption of distinct wavelength bands of light. A smaller bandgap width of the photoresponsive materials results in easy electron transition and a broader range of light wavelength absorption. However, this makes it more susceptible to hole combination.

**Figure 5:**
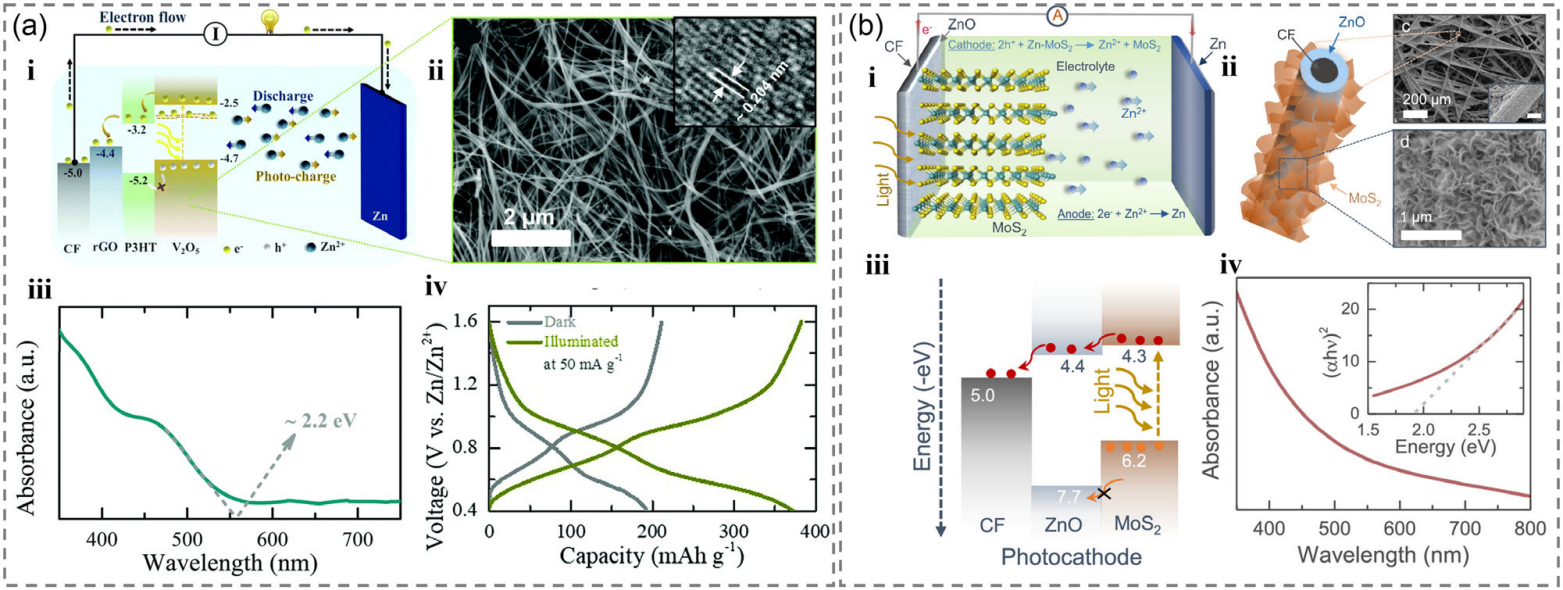
a) i) Photocharging mechanism of photoresponsive zinc‐ion batteries. ii) Scanning electron microscopy (SEM) and iii) UV–vis images of V_2_O_5_ material, and iv) cyclic voltammetry (CV) curves of the battery. Reproduced with permission.^[^ ^58^ ^]^ Copyright 2021, American Chemical Society. b) Photoresponsive zinc‐ion battery of material MoS_2_. i) Photocharging mechanism; ii) SEM images of material MoS_2_; iii) energy band diagram of photoelectrode; iv) UV–vis image of material MoS_2_. Reproduced with permission.^[^ ^59^ ^]^ Copyright 2020, Royal Society of Chemistry.

Vanadium pentoxide (V_2_O_5_), possessing a bandgap energy (≈2.2 eV) suitable for capturing visible light, is a viable candidate as the photoresponsive material of the photoelectrode. The photoresponsive zinc‐ion batteries based on V_2_O_5_ photoelectrodes exhibit a high reversible capacity (≈375 mAh g^−1^) and can conduct charge along the length of the nanofibers, thereby minimizing the recombination before charge extraction and enabling hierarchical photocharge (**Figure** **5**a).^[^ ^58^ ^]^

The MoS_2_ material has a better absorption of the solar spectrum that can improve the overall efficiency of the device. Recently, Michael De Volde et al. used MoS_2_ with smaller bandgap to replace V_2_O_5_ and vanadium dioxide (VO_2_) in the photoresponsive zinc‐ion battery (Figure 5b). MoS_2_ was deposited on ZnO, which was synthesized directly on carbon felt. This research demonstrated the potential of MoS_2_ as a photoresponsive material for improving the power conversion efficiency (PCE) in photoresponsive zinc‐based batteries. The resulting photoelectrodes can be charged by light without any external circuit, providing a higher PCE and a shorter charging time. At a specific current of 100 mA g^−1^ and a light power of 12 mW cm^−2^ at 455 nm, these photoelectrodes can reach a capacity of 245 to 340 mAh g^−1^. The PCE of the photoresponsive battery achieves ≈1.8% using a 455 nm light source and ≈0.2% of PCE in the whole‐sun spectrum.^[^ ^59^ ^]^ In addition, no significant changes in the Zn anode morphology are noticed after 200 cycles, but after 500 cycles, the surface roughness of the Zn anode increased due to zinc dendrite formation. Therefore, the stability of the zinc electrode is also an important part of battery design.

**Figure 6:**
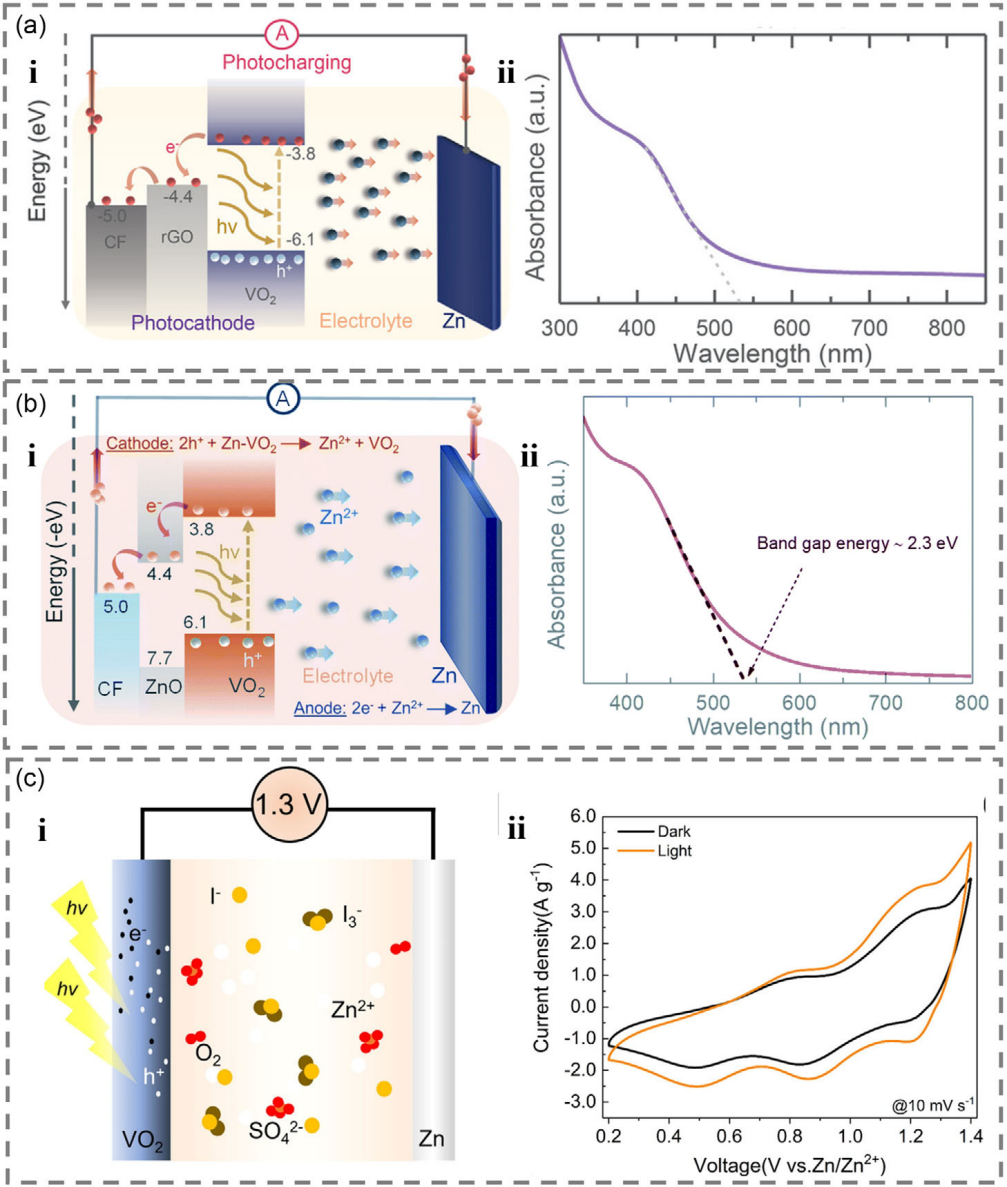
a) i) The photocharging mechanism of VO_2_–rGO photo‐ZIBs and ii) UV–vis images of VO_2_ nanorods. Reproduced with permission.^[^ ^60^ ^]^ Copyright 2021, Wiley‐VCH. b) i) The photocharging mechanismof the VO_2_/ZnO photo‐ZIBs and ii) UV–vis absorption spectrum of VO_2_. Reproduced with permission.^[^ ^61^ ^]^ Copyright 2021, Royal Society of Chemistry. c) i) Schematic of light‐enhanced self‐charging process and ii) CV curves of the photoresponsive battery. Reproduced with permission.^[^ ^62^ ^]^ Copyright 2022, Elsevier.

In the process of photoresponse, the generated photoelectrons will return to the ground state by recombining with corresponding holes, resulting in useless heat and rendering the photoelectrons unsuitable for external circuits. To break this limitation, it is necessary to immediately separate the generated electrons and holes to prevent recombination. The introduction of an electronic transport layer can effectively block the recombination of electrons and holes. For instance, reduced graphene oxide (rGO) is used as an electronic transport layer to transport the excited electrons to the conductive layer and then to the external circuit to generate photocurrent when VO_2_ absorbs photons under illumination to generate excited‐state electrons and holes (**Figure** **6**a), and the PCE of the battery can reach 0.18%.^[^ ^60^ ^]^ Similarly, ZnO achieves the same effect (Figure 6b). The design of battery also uses VO_2_ as the light absorbing material and ZnO as the electron transport layer, which lead to better PCE (0.51%).^[^ ^61^ ^]^

Another strategy to enhance the performance of photoresponsive batteries is optimizing the electrolyte, besides optimizing photoelectrode for the photoresponsive materials. Modification of the electrolyte using polyiodide ions is one such strategy that increases the open‐circuit voltage and capacity while also enhancing the degree of light stimulation (Figure 6c). This phenomenon can be explained by three factors: first, I^−^ spontaneously converts to I^3−^ during charging, which oxidizes HVO_2_ as an oxidant. Second, the presence of multiiodine ions promotes the adsorption of O_2_ and cations, owing to their lone pair electrons and coordination with cations. Finally, the equilibrium potential of I^3−^ relative to Zn/Zn^2+^ is ≈1.3 V, leading to an increase in the open‐circuit voltage of the battery.^[^ ^62^ ^]^

### 3.2 Photoresponsive Zinc–Air Batteries

According to the used electrode number, the architectures of photoresponsive zinc–air batteries can be classified into two systems of two electrodes and three electrodes.

#### 3.2.1 Three‐Electrode System

The traditional photoresponsive batteries generally involve independent photoelectrodes in a three‐electrode system. Therefore, the previous studies of photoresponsive zinc–air batteries with the three‐electrode system mainly focused on the structure design of photoelectrode. Among them, the optimized methods of the photoelectrode could be classified as single‐layer or heterojunction photoresponsive materials, as well as adding hole transport layer.

**Figure 7:**
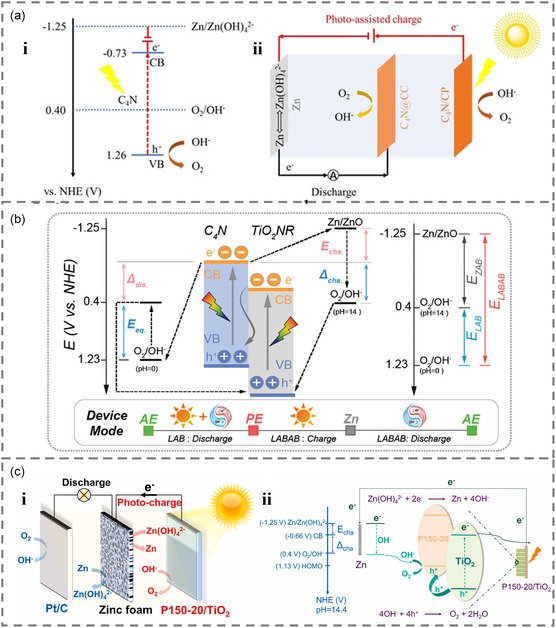
a) i) The operating principle of the photoresponsive battery and ii) proposed working mechanism for VLS‐RZAB. Reproduced with permission.^[^ ^63^ ^]^ Copyright 2021, Wiley‐VCH. b) Light and CNE‐assisted energy conversion/storage mechanism of a C_4_N@TiO_2_NR‐enabled hybrid battery. Reproduced with permission.^[^ ^26^ ^]^ Copyright 2022, Wiley‐VCH. c) The photoresponsive i) Zn–air battery and ii) illustration working mechanism for the charging process of the photoresponsive Zn–air batteries. Reproduced with permission.^[^ ^64^ ^]^ Copyright 2022, Elsevier.

In a typical three‐electrode system, C_4_N can serve as a single‐layer photoresponsive material in the universal photoelectrode to pair with either metal or polymer anode to achieve photoresponsive zinc–air batteries. The mesoporous C_4_N features a narrower bandgap of 1.99 eV with a stronger photocurrent response (**Figure** **7**a). The C_4_N‐based photoresponsive zinc–air batteries delivered good energy storage performance and a low charge voltage of 1.35 V under visible light and the acquired energy efficiency reaches 97.78%, which is superior to conventional rechargeable Zn–air batteries (≈60%).^[^ ^63^ ^]^

On the other hand, the recombination of electrons and holes can be suppressed by designing electron–hole complexes, known as heterojunctions. Hence, the construction of heterojunction is also a smart strategy to enhance the PCE, in particular with S‐type heterojunctions.

In C_4_N, the positively charged carbon sites around pyridinic nitrogen are the most active sites for oxygen reduction reaction (ORR) and OER.^[^ ^63^ ^]^ When C_4_N is brought into contact with TiO_2_NR in the dark to form a heterojunction, electrons spontaneously transfer from C_4_N along the heterojunction interface to TiO_2_NR until their respective Fermi levels reach a balance. This causes negative charges to accumulate on the TiO_2_NR side of the heterojunction region, resulting in a downward bend of CB. Positive charges accumulate on the C_4_N side, causing the VB to bend upward. The C_4_N@TiO_2_NR heterojunction interface generates a strong IEF. Upon illumination, both C_4_N and TiO_2_NR's photoelectrons are excited from the VB to the CB. Under the influence of the IEF and the interface energy band curvature, the photoelectrons on the TiO_2_ CB tend to recombine with the holes on the C_4_N's VB. This retention of electron–hole pairs with strong oxidation–reduction abilities maximizes the photon absorption efficiency (incident photon‐to‐current conversion efficiency: 11.6%) (Figure 7b).^[^ ^26^ ^]^

Moreover, the performance of the photoelectrode can be enhanced by adding a hole‐transport layer. For example, a polymer (P150‐20) nanocoating on TiO_2_ (Figure 7c) can partially suppress oxygen vacancy as a hole trap while simultaneously generating a wealth of superficially active centers to promote the photooxidation reaction. At the lower‐potential boundary, the interface charge transfer/complex ratio (Kt/Kr) is increased.^[^ ^64^ ^]^

### 3.3 Two‐Electrode System

Traditional photoresponsive batteries involve independent photoelectrodes in a three‐electrode, which makes the system bulk, inflexible, and expensive with high‐energy loss.^[^ ^10^ ^]^ Thus, in order to achieve high‐efficiency photoresponsive zinc–air batteries, the internal integration of photoresponsive functionalities into batteries with a two‐electrode configuration is undoubtedly a potential solution, which can allow for direct conversion/storage of the captured solar energy and relieve the energy density concern of the battery. For this purpose, the design of photoresponsive materials has shifted toward the development of dual‐function or even multifunction photoelectrode for a battery with a two‐electrode configuration.

Although the three‐electrode system of dual‐function photoelectrode has been reported,^[^ ^65^ ^]^ the researches on dual‐function or even multifunction photoelectrode were still mainly focused on the two‐electrode system.

Similar to three‐electrode systems, the optimized methods of photoelectrode could be classified as single‐layer or heterojunction photoresponsive materials. Metal oxides (e.g., *δ*‐MnO_2_, NiCo_2_S_4_, BiVO_4_, and *α*‐Fe_2_O_3_) and organic polymers (e.g., pTTh)^[^ 52, 66, 67, 68, 69 ^]^ are common in the development of single‐layer photoresponsive materials. These photoresponsive materials can effectively provide additional photoelectrons for the charge–discharge process. It has the function of promoting OER and ORR, which can reduce the charging voltage and increase the discharge voltage.

Likewise, heterojunctions can be formed at the interface between two semiconductors with different band structures in the photoelectrodes of two‐electrode systems. By combining at least two semiconductors to form a heterojunction, recombination can be effectively suppressed, and even the redox activity of photogenerated carriers can be increased.

**Figure 8:**
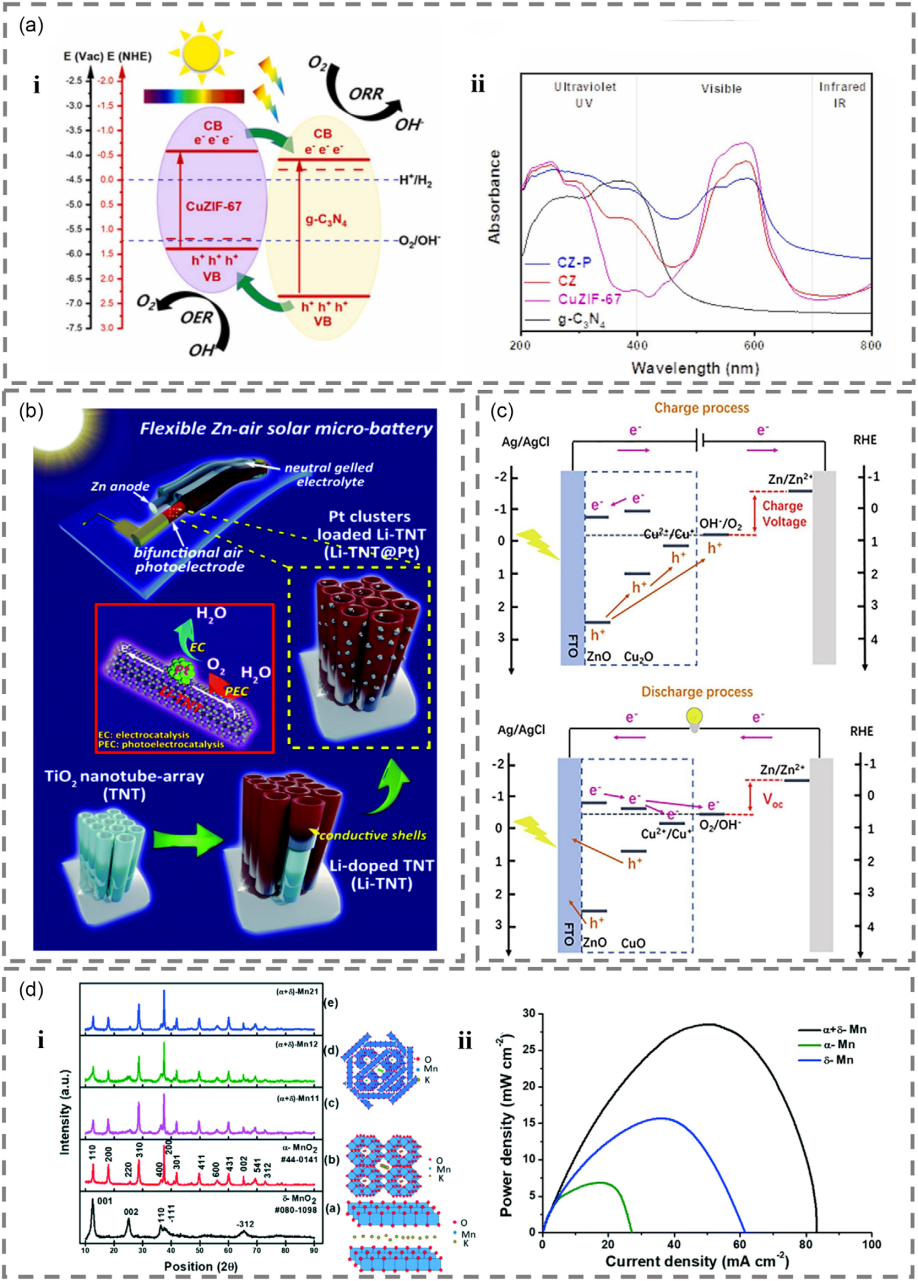
a) i) Working mechanism of CZ. ii) Tauc plots for g‐C_3_N_4_, CuZIF‐67, CZ, and CZ‐P. (Note: CZ‐P: pyrolyzed CZ). Reproduced with permission.^[^ ^70^ ^]^ Copyright 2022, Elsevier. b) Schematic illustration of the preparation route for the air photoelectrode and the assembled solar microbattery. Reproduced with permission.^[^ ^71^ ^]^ Copyright 2022, Royal Society of Chemistry. c) The charge (i) and discharge (ii) mechanisms of the photoresponsive Zn–air battery. Reproduced with permission.^[^ ^72^ ^]^ Copyright 2022, Elsevier. d) i) X‐ray diffraction plots of δ‐MnO_2_, *α*‐MnO_2_, (*α* + *δ*)‐Mn_11_, (*α* + *δ*)‐Mn_12_, and (*α* + *δ*)‐Mn_21_. ii) Power density curves of the zinc–air battery. Reproduced with permission.^[^ ^73^ ^]^ Copyright 2020, Royal Society of Chemistry.

For instance, g‐C_3_N_4_/CuZIF‐67(CZ) could be a dual‐function air electrode with an appropriate bandgap (1.99 eV), which ensures efficient light absorption across the UV–vis–NIR region (**Figure** **8**a), promoting the participation of more photoelectrons in the reaction. Moreover, the bandgap structure and the BEF at the p–n junction of CZ enhance the separation efficiency of electron–hole pairs, minimize overpotential losses, and ultimately improve the battery performance.^[^ ^70^ ^]^

Another example is the dual‐function photoelectrode composed of Li‐TNT@Pt, Zn‐deposited TiO_2_ wire as the anode electrode, and neutral gel electrolyte. In the design of the photoresponsive materials, a 2–3 nm amorphous Li‐doped TiO_2_ layer was found to be situated between TiO_2_ and Pt electrode. This layer not only significantly improves charge transfer, but also modifies the electronic structure of Pt. Thus, the activity of OER and ORR were enhanced (Figure 8b), and energy efficiency reached 90%–110% (some input energy comes from solar illumination).^[^ ^71^ ^]^

Besides, ZnO/CuO is a dual‐function material derived from copper valence state changes (Cu_2_O and CuO). Researchers have employed 3D printing technology to produce physical models of batteries, offering a novel approach to battery design. The battery they have designed exhibits a charging and discharging voltage of 1.5 V/1.28 V (traditionally 2 V/0.63 V) (Figure 8c) and PCE is 0.3% with 0.8 mA cm^−2^, representing a significant breakthrough for zinc–air batteries with copper photoelectrode.^[^ ^72^ ^]^

Bifunctional electrodes in rod‐shaped form, such as the tunnel structure of *α*‐MnO_2_ and the 2D *δ*‐MnO_2_, have also been studied due to their high specific surface area and layered structure advantages, resulting in composite materials with power density about 2–4 times higher than that of traditional structure materials (Figure 8d).^[^ ^73^ ^]^

To sum up, these materials based on heterojunctions in photoresponsive zinc–air batteries can be divided into the types of inorganic–inorganic, organic–organic, organic–inorganic hybrids, etc.

**Figure 9:**
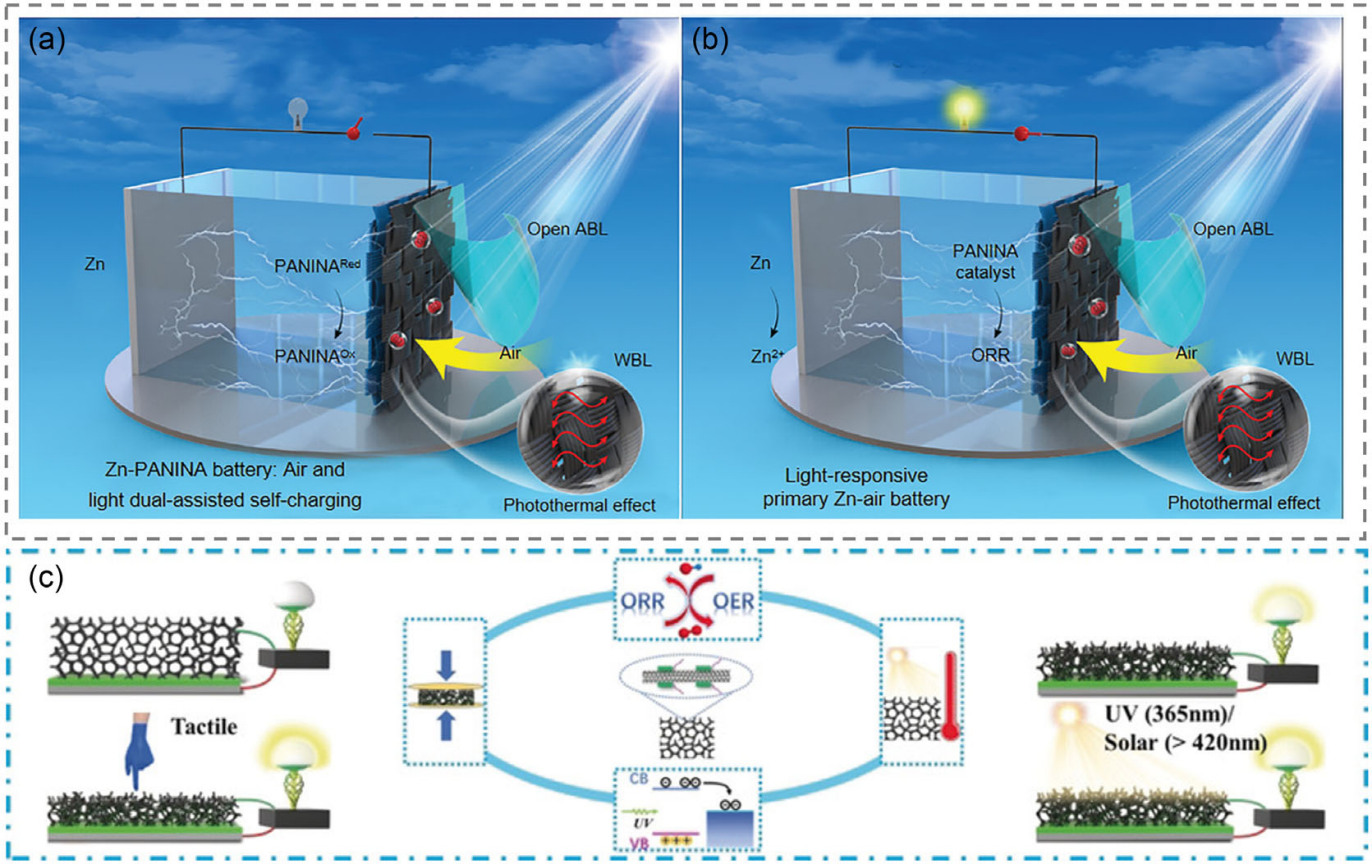
a) Air and light dual‐assisted self‐charging Zn–PANINA battery. Reproduced with permission.^[^ ^74^ ^]^ Copyright 2020, Wiley‐VCH. b) Photoresponsive primary Zn–air battery. Reproduced with permission.^[^ ^74^ ^]^ Copyright 2020, Wiley‐VCH. c) Multisensing rechargeable batteries. Reproduced with permission.^[^ ^75^ ^]^ Copyright 2019, Wiley‐VCH.

Notably, the design of multifunction photoelectrode for photo‐responsive batteries has attracted increasing interest more recently. Especially for photothermal effect, the efficient utilization of photothermal effect can effectively improve the energy absorption of long‐wavelength sunlight, resulting in reduction of the energy loss caused by heat dissipation. Yu et al. used the photothermal effect successfully to increase the specific capacity of a photoresponsive zinc–air battery from 379.4 to 430 mAh g^−1^ (**Figure** **9**a,b).^[^ ^74^ ^]^

Moreover, the multiresponse design of the battery (pressure, photothermal, and photoelectric) opens a new direction for the future development of photoresponsive batteries. For example, multiscale PEDOT‐PEO‐CNTs‐PUF can serve as all‐in‐one air electrodes combining bifunctional ORR/OER activities, piezoresistance effect, photothermal effect, and photoelectric conversion function, which enable effective modulation of interfacial properties, electronic and ionic transport, or redox reactions (Figure 9c).^[^ ^75^ ^]^

## 4 Conclusion and Perspective

A multitude of design strategies can be utilized to develop photoresponsive zinc‐based batteries that enable cost‐effective and efficient utilization of solar energy. These batteries can improve energy conversion and storage efficiency, thus addressing the energy storage and density problems faced by conventional batteries and solar cells. Significantly, the exceptional photoresponse of these batteries makes them highly suitable for next‐generation smart electronics, photovoltaics, and sensors.

Photoresponsive zinc‐based batteries have made some progress, but there are still many challenges to overcome. In a photoresponsive battery, the performance of the photoelectrode is the most important factor. The most direct way is to improve the bandgap of the photoelectrode in order to enhance its ability to absorb sunlight. Photoresponsive materials with suitable bandgap and modification are the basis of improving the absorption of sunlight. In addition, reasonable design of the photoelectrode structure, such as the construction of heterojunctions, the design of hole‐transport layers, or electron‐transport layers, can more effectively improve the separation and transport capacity of hole–electron pairs, thereby avoiding carrier recombination. Moreover, multiresponse cathode materials are also a future trend. In addition to photoelectric effect, other responses, such as photothermal effect, allow batteries to adapt to complex working conditions.

Second, the stability of the battery is another important property of photoresponsive zinc‐based battery. The main causes of battery instability are dendrites generated by the zinc electrode, uneven distribution of zinc electrode current, and electrolyte corrosion, which are the major problems that need to be addressed. Hence, the rational design of zinc electrode should aim to make the current generated during discharge evenly distributed. On the other hand, the development of solid/quasisolid electrolytes is another solution that can greatly improve battery stability, compared to the corrosiveness and volatility of liquid electrolytes. However, little research has been conducted on photoresponsive solid/quasi‐solid zinc‐ion batteries.

Finally, in addition to optimization of photoresponsive materials and device structures, the standard method used to measure the performance of the battery is also extremely important. Currently, there is no standard set of measures and the parameters used in measuring the performance varies. Using standardized measures to accurately compare the performance of different systems will further confirm operating mechanism and application potential of photoresponsive batteries. The development of photoresponsive zinc‐based batteries would promise a bright future for solar energy. Further expanding the potential of energy conversion and storage in battery systems is a promising research direction. It is anticipated that more and more promising research will be conducted on photoresponsive zinc‐based batteries, ultimately leading to their commercial application.

## Conflict of Interest

The authors declare no conflict of interest.
